# Does the age at disease onset cause a delay in diagnosis and affect disease severity in the children with familial Mediterranean fever?

**DOI:** 10.1186/1546-0096-13-S1-P75

**Published:** 2015-09-28

**Authors:** O Kuru, O Ozkaya, G Alayli, G Genc, D Durmus, A Bilgici, HE Sen

**Affiliations:** 1 Ondokuz Mayis University, Medical Faculty, Physical Medicine and Rehabilitation, Samsun, Turkey; 2 Ondokuz Mayis University, Medical Faculty, Pediatric Nephrology, Samsun, Turkey

## Introduction

Familial Mediterranean fever (FMF) is a systemic autoinflammatory disease characterized by recurrent attacks of fever and sterile peritonitis, pleuritis, arthritis or erysipelas-like erythema. Symptoms related with FMF can begin at very early ages of life. However, since most symptoms related to FMF are often well-known only when children become more verbal, and fever in children in the first years of life are common, attacks of fever alone may cause or contribute to diagnosis delay.

## Objectives

In the present study we aimed to investigate whether the age at onset of the symptoms cause a diagnosis delay in the children with FMF.

## Materials and methods

Ninety FMF subjects (M/F: 43/47) who fulfilled the Livneh criteria for the diagnosis of FMF were enrolled into the study. Subjects were between 8-18 years age and having the symptoms of FMF for at least one year. Demographic data including age, age at onset of the symptoms, duration of symptoms and family history were recorded. The subjects were questioned about periodic fever, abdominal and chest pain, arthritis, and erysipelas-like erythema. The severity score of the disease was calculated based on the Tel-Hashomer severity score. 6 Minute Walk Test (6MWT) was used to evaluate functional capacity.

## Results

The mean age of the FMF subjects was 12.21±2.75 years. Abdominal pain attacks were the most common symptom, occurring in 77 (85.6%) of our patients; periodic fever was in 74 (82.2%), chest pain in 36 (40%), arthritis in 41 (45.6%), and erysipelas-like erythema in 12 (13.3%). A positive family history for FMF was detected in 51 (56.6%) of the patients. Onset age and diagnosis delay duration did not significantly differ between the groups regarding family history (p>0.05). Twenty-one children (23.3%) had the first FMF symptoms at the age of <5 years. While the mean onset age was 6.91± 3.44 years, delay in diagnosis was 2.12±2.70 years. In the children with FMF, while there was a negative correlation between the age at onset of the symptoms and duration of delay and disease severity score, there was no correlation between 6MWT.

## Conclusion

We determined that FMF beginning at early ages causes a delay in diagnosis and also a worse disease severity score. Physicians practice in the regions that FMF is prevalent should be aware of different features of the disease in childhood in order to reduce a possible delay in diagnosis and prevent the development of complications.

